# Dementia education programs for Chinese, Japanese, Korean, Filipino, and Vietnamese Communities: A scoping review

**DOI:** 10.1002/alz70860_101583

**Published:** 2025-12-23

**Authors:** Kexin Yu, Maria Phung, Jiaming Liang, An Nguyen, Tamima Rahman, Gabriela E Caballero, Ann Dadich, Michelle DiGiacomo, Genevieve Z Steiner‐Lim, Rema Raman, Joyce Siette, Diana Karamacoska

**Affiliations:** ^1^ University of Texas Southwestern Medical Center, Dallas, TX, USA; ^2^ Western Sydney University, Sydney, NSW, Australia; ^3^ University of Texas Health Science Center at Houston, Houston, TX, USA; ^4^ University of Technology Sydney, Sydney, NSW, Australia; ^5^ Alzheimer's Therapeutic Research Institute, University of Southern California, San Diego, CA, USA

## Abstract

**Background:**

Asian immigrants can experience health disparities and inequities due to language barriers and the limited availability of culturally appropriate services. Dementia education is needed to combat dementia misperceptions and promote help‐seeking. This scoping review describes the characteristics and impacts of community‐based dementia education programs for large East and Southeast Asian immigrant groups worldwide, including Chinese, Japanese, Korean, Filipino, and Vietnamese communities.

**Methods:**

Publications in the English language from the last two decades were identified through a database search of Scopus, CINAHL Plus, PsycInfo, Embase, and OVID (MEDLINE and Emcare). We included education programs for community‐dwelling Asian adults and people with dementia and their carers. Studies conducted in both the immigrant origin and destination countries were included as education programs in both contexts might address shared health beliefs and practices. Educational programs for clinical workforces or professional development were excluded, given that workforce building is the focus of this review.

**Results:**

The search identified 3,431 publications. Twenty‐seven articles (17 in Asian countries, 8 in the USA, 2 in Australia, *n* = 10‐4,333) met the inclusion criteria: 12 for community members, and 15 for carers. Six involved people living with dementia or carers in program design. Programs for community members were delivered via group meetings in‐person or online, as well as with YouTube videos and virtual reality experiences. Two publications addressed cultural beliefs towards aging and dementia. Many educational programs for community members demonstrated improvement in health literacy. Dementia education programs for carers tended to be structured. Two of the 15 carer programs involved cultural adaptation. Carer programs reduced carer burden, improving skills and self‐efficacy. Most programs were conducted with Chinese, Korean, Japanese, and Vietnamese populations, one included Filipino community members, and no carer programs involved Filipino carers.

**Conclusions:**

Educational programs can be successful, but need more cultural specificity, and most were at the pilot‐testing stage. Publications on programs for Filipino communities were scarce compared to other Asian communities. Future reviews should include publications that are not in English and those on South Asian immigrants.

